# Novel Humanitarian Aid Program: The Glivec International Patient Assistance Program—Lessons Learned From Providing Access to Breakthrough Targeted Oncology Treatment in Low- and Middle-Income Countries

**DOI:** 10.1200/JGO.2015.000570

**Published:** 2015-09-23

**Authors:** Pat Garcia-Gonzalez, Paula Boultbee, David Epstein

**Affiliations:** **Pat Garcia-Gonzalez**, The Max Foundation; **Paula Boultbee**, PTB Consulting LLC, Seattle, WA; and **David Epstein**, Novartis Pharmaceuticals, East Hanover, NJ.

## Abstract

Imatinib was the first targeted therapy approved for the treatment of cancer. With its approval, it was immediately clear to Novartis that this breakthrough therapy would require an innovative approach to worldwide access, with special consideration of low- and middle-income countries. Lack of government reimbursement, universal health care, or health insurance coverage, few trained specialty physicians or diagnostic services, and poor health care infrastructure were, and continue to be, contributing barriers to access to treatment in low- and middle-income countries. The Glivec International Patient Assistance Program (GIPAP) is an international drug donation program established by Novartis Pharma AG and implemented in partnership with The Max Foundation, a nonprofit, nongovernmental organization. GIPAP was established in 2001, essentially in parallel with the first approval of imatinib for chronic myeloid leukemia. Since 2001, GIPAP has made imatinib accessible to all medically and financially eligible patients within 80 countries on an ongoing basis as long as their physicians prescribe it and no other means of access exists. To date, more than 49,000 patients have benefited from GIPAP, and 2.3 million monthly doses of imatinib have been approved through the program. GIPAP represents an innovative drug donation model that has set the standard for access programs for other targeted or innovative therapies. The purpose of this article is to describe the structure of GIPAP, as well as important lessons that have contributed to the success of the program. This article may assist other companies with the development of successful and far-reaching patient assistance programs in the future.

## INTRODUCTION

Chronic myeloid leukemia (CML) is a hematologic malignancy that annually affects between one and 1.6 of 100,000 people worldwide.^1^ One of the rarest forms of leukemia in terms of annual incidence, CML is expected to become the most prevalent hematologic malignancy in the world by 2020.^2–4^ Once an acutely life-threatening condition with a median survival of often fewer than 5 years,^2,3^ CML has been considered a chronic illness since the advent of targeted therapies.^5–9^ Imatinib (Glivec, Gleevec; Novartis Pharmaceuticals, East Hanover, NJ) was the first targeted therapy to be developed and approved for the treatment of a cancer. Imatinib was approved for the treatment of accelerated- and blast-phase Philadelphia chromosome (Ph) –positive CML in May 2001 in United States and for the first-line treatment of Ph-positive CML in January 2002 in both the United States and Europe. It was subsequently approved for the treatment of GI stromal tumor (GIST) in 2002.^5^

Despite advances in the treatment of cancer and other diseases like HIV, hepatitis C virus, and malaria, patient access to lifesaving medications in low- and middle-income countries (LMICs) is often challenging for both financial and logistic reasons.^10,11^ Lack of reimbursement or health insurance coverage, inadequate infrastructure, poor access to trained specialty physicians or diagnostic services, and insufficient patient health education are all contributing factors to poorer patient outcomes in LMICs.^12^ It is estimated that in many LMICs, more than 80% of pharmaceuticals must be purchased by patients directly (private payer systems).^13,14^ The international community has responded to this unmet need by establishing humanitarian donation programs of varying size and scope. There are many examples of such donation programs that have historically fallen prey to organized theft and corruption, leading to the illegal selling of often substandard medicines on the black market; in other instances, these medicines have been mishandled and rendered ineffective and have ultimately never made it to the intended patient population.^15^ These challenges faced by the international community have led to the development of manufacturer-led international donation programs as well as WHO guidelines for drug donations to ensure broad and equitable distribution of lifesaving therapies to patients in need worldwide.^16^

**Figure 1:**
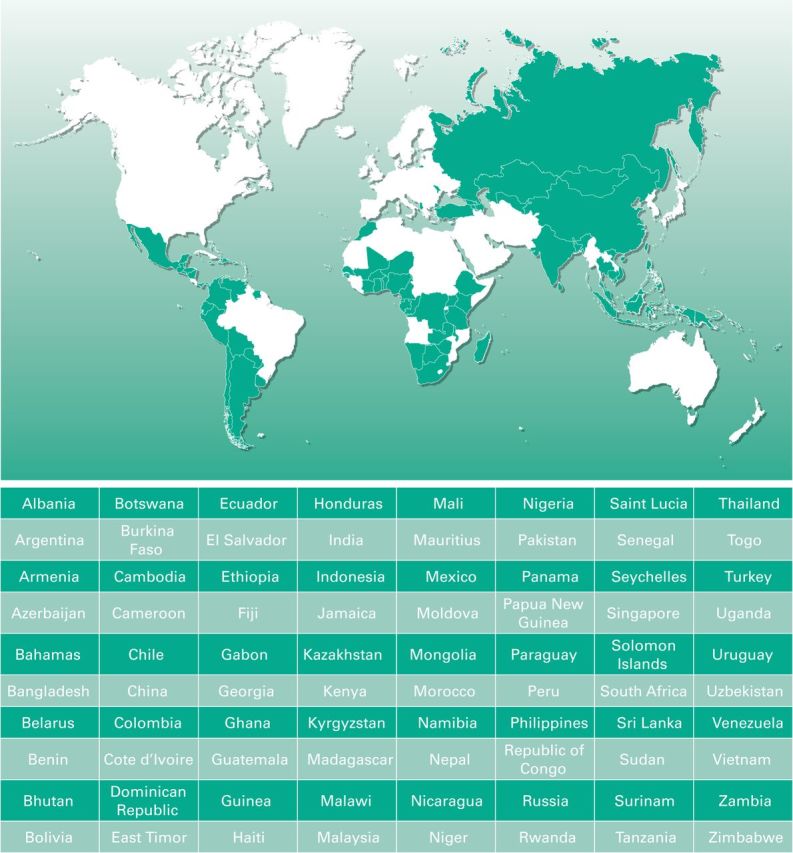
Glivec International Patient Assistance Program country map and list.

The Glivec International Patient Assistance Program (GIPAP) is an international donation program established by Novartis Pharma AG in partnership with The Max Foundation, a nonprofit, nongovernmental organization (NGO) that serves as the program administrator.^16^ The remit of the program is to facilitate access to and distribution of imatinib to patients in LMICs, who meet specific program criteria. Following the WHO guidelines for drug donations,^17^ Novartis provides imatinib at no cost to eligible patients with CML or GIST, the approved indications for the drug, in participating countries. Eighty LMICs in Africa, Asia, Eastern Europe, Latin America, and the Caribbean that have no comprehensive drug reimbursement system or available generics have participated in GIPAP. To date, 49,477 patients from 80 countries (Patient Assistance Tracking System [PATS database]) have benefited from GIPAP since the inception of the program in late 2001 (Fig 1), and more than 2 million 1-month supplies of treatment have been approved for donation.^18^

The development of a drug donation program for a targeted cancer therapy was uncharted territory in 2001. The path to the growth of this program was peppered with successes but riddled with major hurdles along the way. Each hurdle led to key learning and subsequent adjustments that helped the program mature and evolve into a robust example of best practice and the most comprehensive and far-reaching access program developed for an oncology drug, one that to this day still profoundly affects patients and communities worldwide. The purpose of this article is to describe the partnership between the donor company, Novartis, and The Max Foundation, the NGO that administers the program, as well as to describe the current structure of GIPAP, highlighting the novel characteristics that were implemented by trial and error, to enable others to draw parallels and benefit from this collective experience.

## HISTORY AND INITIATION OF GIPAP

The GIPAP partnership between Novartis and The Max Foundation was initiated in late 2001, at a time when imatinib had been newly approved for CML based on the results of the IRIS (International Randomized Study of Interferon Versus STI571) trial.^19^ Immediately after the first approval of imatinib, it was clear to Novartis that many patients worldwide would benefit from access to this therapy. Still, given the novel type of cancer therapy, one allowing patients to be treated on an outpatient basis but requiring chronic treatment and regular monitoring indefinitely, there were many difficult questions to answer before embarking on a drug donation initiative. For example:

- How many patients would be properly diagnosed and thus eligible for a patient assistance program, and what infrastructure would be required to fairly provide imatinib to these patients consistently?
- How could a patient assistance program ensure that requests for imatinib would come only from hematologists and oncologists with experience and qualifications to safely monitor their patients' treatment?
- How could this program ensure that only appropriate, eligible patients gained access to imatinib? Given the robust global press coverage of imatinib, including a *Time* magazine cover (May 28, 2001) hailing it as a magic bullet for cancer, might some eager health care providers attempt to treat other cancers with the donated drug, possibly endangering the lives of their patients with cancer?
- How would Novartis identify a suitable partner to administer the imatinib drug donation program?

The model proposed by Novartis for this donation program required identifying individual diagnosed patients and guiding them through the program. This led Novartis to the decision to seek an NGO with a patient-focused approach. The search for a partner that could serve as the administrator of the program was a critical step in implementing GIPAP and beginning to address these challenges. Large organizations with a focus on cancer as well as organizations that focused on hematologic malignancies specifically were considered. Novartis sought a unique NGO with a vested interest in specifically fighting CML, because focus on and awareness of this rare disease were minimal among other larger foundations with broader interest in hematologic malignancies or cancers experienced by a larger number of patients. The Max Foundation, founded in 1997 in the memory of Maximiliano (Max) Rivarola, who tragically succumbed to CML at the age of 17 years, was chosen not only for its clear and pointed focus on CML, but also because of its grassroots efforts and strong emotional connection to patients with CML. The organization had a deep commitment to the betterment of patients and a global scope with focus on LMICs. For these reasons, The Max Foundation was an obvious choice as partner in the GIPAP initiative, but its small size and relatively new standing in the NGO landscape presented challenges. The Max Foundation was faced with the herculean effort of administering an international donation program for a novel, targeted cancer therapy. This included identifying and verifying the identity and eligibility of patients across many regions of the world and liaising with health care providers for each specific patient, including those in regions where Internet access and communications were extremely limited. The logistics of launching a donation program of this scope could be daunting. For example, because donated medicines maintain high market values, which may result in high import taxes, tax exemptions must be considered and obtained in each country.

Despite these major hurdles, the GIPAP partnership was formally established in 2001, just months after US Food and Drug Administration approval of imatinib for CML.^20,21^ Just as imatinib was the first targeted therapy for CML, GIPAP was the first patient assistance program for a targeted therapy, making The Max Foundation and Novartis pioneers in this field. Since its launch, GIPAP has expanded its reach from a total of 13 patients approved in 2001 to more than 49,000 patients by 2015, with more than 20,000 of them still actively receiving donated product (Table 1). The scope of GIPAP as a function of time includes approximately 2,340,000 months of imatinib treatment approved for donation to date (PATS database).

**Table 1:** GIPAP Patient and Physician Approvals

Year	GIPAP Patients by Disease Type (n = 49,484)			No. of GIPAP Physicians (n = 1,502)
	CML	GIST	Other*	
2001	13	—	—	†
2002	740	32	—	†
2003	2,146	130	—	†
2004	4,705	387	—	†
2005	4,335	583	—	205
2006	5,264	1,021	—	142
2007	5,863	1,426	17	171
2008	5,672	1,398	41	158
2009	2,136	454	37	46
2010	1,676	404	63	38
2011	1,827	502	67	42
2012	1,815	588	62	33
2013	1,838	616	66	53
2014	1,825	644	95	32
2015‡	688	239	33	22
Total	40,577	8,426	481	1,502

Abbreviations: CML, chronic myeloid leukemia; GIPAP, Glivec International Patient Assistance Program; GIST, GI stromal tumor.

* Other includes: Philadelphia chromosome–positive acute lymphoblastic leukemia, myelodysplastic and myeloproliferative diseases, systemic mastocytosis, hypereosinophilic syndrome and/or chronic eosinophilic leukemia, and dermatofibrosarcoma protuberans.

† Data not available in annual increments before 2005. Total No. of physicians from 2001 to 2004 was 560.

‡ As of May 25, 2015.

## GIPAP MODEL

What differentiates GIPAP from other large international drug donation efforts is its direct-to-patient approach. The Max Foundation identifies and verifies the eligibility of each individual patient and requests a product donation from Novartis at a specific dose as prescribed by that patient's physician in 3-month intervals. In this way, the organization ensures that only properly diagnosed, eligible patients gain access to imatinib. The organization carefully tracks the time and consumption need for resupply of each patient. The Max Foundation plays a key role in the protection of the privacy of patients by assigning a unique identifier to each patient to be used in all communications with Novartis.

In setting up GIPAP, and as the program owner, Novartis is tasked with identifying the appropriate countries in which to implement the program and establishing the medical and financial eligibility criteria. Although there were no direct-to-patient donation programs before GIPAP, the WHO guidelines for drug donations have provided the basic foundation for the medical criteria of the program, which accepts only patients with a confirmed diagnosis of an approved indication for imatinib.^17^

Novartis is responsible for delivering the drug to the treating cancer institution at the requested dose and amount for each specific patient, labeling imatinib supply for GIPAP with a “for patient assistance” sticker to deter product diversion and support appropriate use, as well as to ease the process of obtaining tax waivers. In countries where Novartis does not have the necessary infrastructure to support drug importation, the company contracts with Axios International to aid in these logistics. The selection of qualified institutions and physicians is performed in collaboration between Novartis and The Max Foundation.

Participation of physicians and nurses in GIPAP is voluntary, but their commitment has greatly contributed to the successful treatment of their patients. The physicians and nurses at GIPAP sites comply with the demanding data entry needs of the program, in many cases support importation and tax exemption processes, and personally contact patients with appointment reminders and other important information. These interactions foster strong peer-to-peer support and patient group development. As the program has ultimately helped increase the prevalence of CML in these communities, the number of living patients with CML under the care of each physician has grown dramatically in the program.

**Figure 2:**
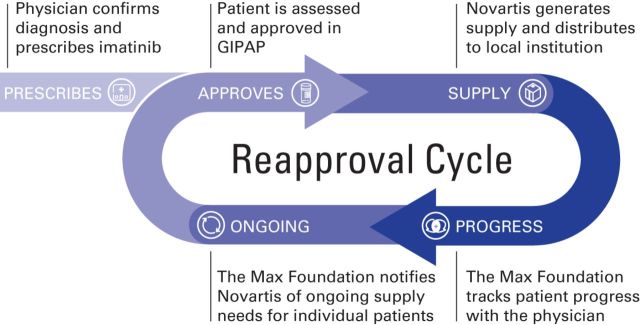
Glivec International Patient Assistance Program (GIPAP) drug donation flow.

The company, the program administrator, and the physician work in close communication to ensure that each patient receives needed product on a periodic basis without interruptions in treatment (Fig 2). This is a critical aspect of this new model, because identifying each individual patient and delivering drug in accordance with specific need prevent drug stockpiling. A donation program such as GIPAP requires a significant investment of resources from the donor company and can have an important impact on drug production. As an example, in 2014, approximately 30% of all imatinib produced by Novartis was for donation, with a large part of it supporting GIPAP (data on file, Novartis, East Hanover, NJ).

A challenge of donating tyrosine kinase inhibitors such as imatinib is the chronic and outpatient nature of this treatment. It has been shown that achieving optimal clinical outcomes for patients receiving imatinib is strongly linked to patient adherence to treatment.^22–25^ As a patient-focused organization, The Max Foundation is able to offer direct support services to patients in GIPAP across many countries.^16^ These support services, although not part of the program itself, holistically contribute to the successful long-term participation of patients in GIPAP and are vital to the environment within which the program operates. Collectively, for The Max Foundation, the average number of one-on-one patient contacts per month in 2013 was more 10,000 across countries.^18^

## IMPACT OF GIPAP PARTNERSHIP ON CREATION OF SUSTAINABLE LOCAL RESOURCES

To further provide support services to patients with CML and GIST, over the past 14 years, The Max Foundation has diligently worked to identify local patient leaders in the GIPAP countries and together with them has embarked on the establishment of patient organizations. In partnership with these patient organizations, The Max Foundation provides peer-to-peer support, facilitating disease education and supporting adherence to prescribed therapies. The Max Foundation has been directly engaged in the establishment of 35 local patient support organizations and currently supports a total of 68 organizations in 58 countries through the Max Global Network.^16^

**Figure 3:**
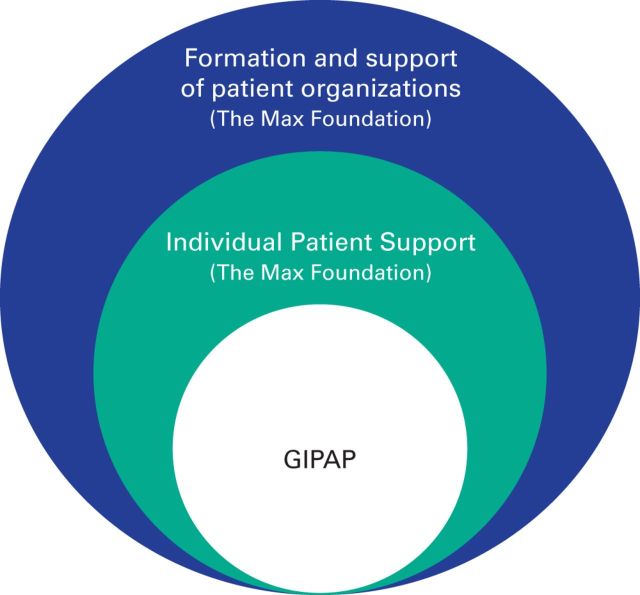
The Max Foundation patient assistance and support model. GIPAP, Glivec International Patient Assistance Program.

Novartis, along with other companies with interest in CML and GIST, continues to support educational initiatives and awareness campaigns initiated by The Max Foundation and its partner organizations. One such global campaign entitled “What is my PCR?” helps both physicians and patients better understand a diagnosis and how best to optimally manage their disease.^26^ These independent elements collectively contribute to the success of GIPAP (Fig 3).

## CASE STUDY IN INDIA

India has the largest number of GIPAP recipients (12,668 patients helped), representing 25% of all program recipients, and has been an active GIPAP country since 2002. To support physicians and help patients navigate the program verification and approval process in India, The Max Foundation expanded its team to include a local cancer care provider in 2002 and formally established the first India MaxStation. MaxStations, as designated by The Max Foundation, are local advocates with experience in cancer support that facilitate communications and data transfer among all program stakeholders. MaxStations are a core component of the internal structure of The Max Foundation. Outside of their role as GIPAP program administrators, all MaxStations dedicate 20% of their time to providing support services to patients. In May 2002, when the first MaxStation was incorporated in India, there were 22 active patients in GIPAP in the country. One year later, with 620 patients active in GIPAP in India, The Max Foundation launched a new patient support initiative, Friends of Max, in partnership with local patient leaders. Friends of Max was launched in five cities in May 2003 as the patient support arm of The Max Foundation in India. Through Friends of Max, The Max Foundation provides disease education, antistigma campaigns, adherence programs, and other support initiatives to patients with CML and GIST in India.

In the past 14 years, The Max Foundation has established MaxStations in 13 countries to support GIPAP. Furthermore, the organization has engaged with patient organizations, treating physicians, and patient leaders in other GIPAP countries to support the delivery of support services around patients.

## LESSONS LEARNED FROM GIPAP

- It is possible to safely and consistently deliver targeted therapies to patients in any region of the world.

**Figure 4:**
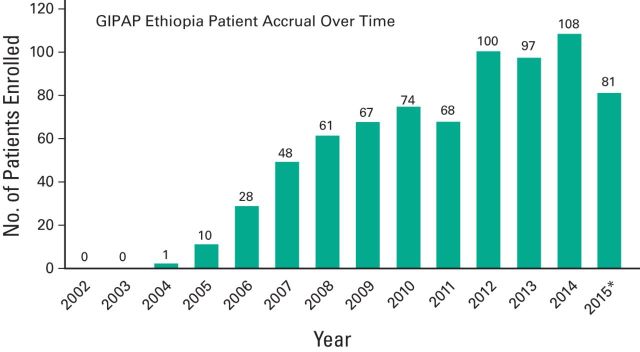
Glivec International Patient Assistance Program (GIPAP) Ethiopia patient accrual over time. (*) As of June 1, 2015.

Through GIPAP, it has been demonstrated that with the right partnerships and true commitment of all parties, it is possible to safely provide access to targeted cancer treatment for patients in any corner of the world. Moreover, in countries with limited resources to treat a specific cancer, starting with a commitment to access to therapies can be a catalyst to improving the health care infrastructure necessary to treat the disease, as well as to developing sustainable local resources such as patient support organizations. For example, access to molecular monitoring, a core component of effective CML therapy, was essentially nonexistent in most GIPAP countries in 2001. Access to imatinib through GIPAP led The Max Foundation, Novartis, and others with interest in CML to develop initiatives to improve access to reliable diagnostics in LMICs. In 2011, the first molecular test to measure *BCR*-*ABL* in Sub-Saharan Africa was performed in Black Lion Hospital, Addis Ababa, Ethiopia. Accessibility of diagnostics had a profound impact on the success of the program, with an increase of 30% in the annual accrual of patients in Ethiopia (Fig 4). Today, similar resources are available for diagnosing and monitoring CML in many GIPAP countries.

- Forecasting drug supply and complying with pharmacovigilance regulations are key challenges of the model.

Assessing the drug supply needs of each country and providing adequate supply in a timely fashion to all patients have been major challenges throughout the implementation of GIPAP. Time for production and importation must be taken into account, a process that ranges from 6 to 8 weeks depending on the location. The likelihood of changes in laws and personnel regulating importation of donated products in LMICs poses threats to the capacity of the program partners to prevent treatment interruptions. Although certain circumstances are unavoidable, a lesson learned from the GIPAP experience is the need to engage and educate local stakeholders regarding the initiative to ensure an efficient drug importation process and day-to-day running of the program. This process has evolved since the inception of GIPAP. When GIPAP was initially established, there was an urgency to provide potentially lifesaving therapy to seriously ill patients without options as soon as possible. Thus, the program was initiated as what could be perceived as a vertical donation program, and there was little time available to engage with key local stakeholders broadly. With the maturity of the program, a concerted effort has been made to attend to the role of important local stakeholders who can help facilitate the smooth and consistent implementation of the program long term.

Furthermore, pharmacovigilance, although a most necessary practice to protect the safety of patients, is one of the most challenging aspects of the GIPAP model. Pharmacovigilance is a requirement of all health authorities to ensure the safety of patients receiving treatment, and companies are thus required to report all adverse events that occur in their patient-oriented programs, including any drug donation program. As a proxy of the company, The Max Foundation team must report all adverse events of which they become aware occurring in GIPAP. GIPAP captures a high volume of adverse events thanks to the large number of participants, their length of time receiving treatment, and regular interaction by The Max Foundation with people in the program. The Max Foundation had to provide a solution to manage this significant and important administrative burden. To do so, The Max Foundation expanded its staff and quickly developed the necessary structures for tracking all adverse event reports. PATS, developed by The Max Foundation to support and coordinate all organizational activities in GIPAP, today includes an adverse event reporting tool that ensures high-quality and timely reporting. While complying with the mandates of safety reporting, The Max Foundation continues to protect patient confidentiality by submitting the reports using the personal unique identifiers assigned to each patient. The Max Foundation is required to report the events to the Novartis safety team, which must work with each treating physician to complete the information required and subsequently submit it to the health authorities as per regulatory requirements. Perhaps the heaviest burden of this process lies with the health care providers. Because GIPAP operates in the real world, and physicians are often overburdened, the addition of this requirement is yet another stress on their time and resources.

PATS is a smart Web engine that also serves to propel the life cycle of patients in the program, a feature without which the program would not succeed. With case-by-case management capacity, PATS alerts The Max Foundation team of the time to start the resupply process for each patient. Moreover, the system allows health care providers to input needed information regarding each patient in real time to generate resupply requests to Novartis. PATS thus facilitates real-time logistics along with facilitating pharmacovigilance.

- Drug donation and patient assistance programs must be tailored to unique and changing country-specific needs.

One of the challenges of establishing a long-term drug access program is being able to evolve over time as the natural environment changes. Some countries, where a program like GIPAP made sense in 2001, presented different characteristics a decade later. In 2009, Novartis unveiled the Novartis Oncology Access (NOA) program as an expansion of GIPAP. NOA has been implemented in countries where factors such as a growing middle class, development of universal health care systems, or otherwise improved affordability of medicines have created the opportunity to engage local stakeholders in sharing the responsibility of providing access to imatinib. Under NOA, Novartis shares the cost of imatinib either with government health care systems, charities, and other payers or directly with patients without health care coverage who are unable to pay for the full cost of their medication but can pay it in part under a copay model. In some countries such as India, a third-party financial institution conducts the financial assessment of patients, while working closely with The Max Foundation to ensure continued support from the broad physician network, patient engagement services, and uninterrupted delivery of medicines. Since its launch in 2009, NOA India has further expanded access to imatinib by providing treatment to 17,000 additional patients (PATS database).

- GIPAP improves our understanding of disease in LMICs.

Important information about the diagnosis and treatment duration of patients with rare diseases in LMICs may be gleaned through programs like GIPAP. In a recent analysis of GIPAP data, the age at diagnosis was shown to be significantly lower among patients treated with imatinib in LMICs; a correlation was also demonstrated between the time from diagnosis to treatment with overall survival rates in those countries.^27^ Since time from diagnosis to tyrosine kinase inhibitor treatment is a variable that can be improved through education and access to proper diagnostics, this analysis helped identify an area that could be independently targeted to improve patient outcomes. Another recent analysis of GIPAP data demonstrated that the outcomes of pediatric patients with CML treated with imatinib in LMICs, an area poorly understood previously, are similar to those of pediatric patients in the United States, as long as access to therapy is provided promptly and consistently.^28^ Similar findings were demonstrated with adult patients in GIPAP.^29^ Factors that contribute to successful GIPAP enrollment and patient outcomes within participating GIPAP sites have also been analyzed. It was found that GIPAP sites with at least one hematologist/oncologist and with diagnostic and research capabilities were associated with higher patient enrollment numbers and better patient outcomes versus sites without one of these factors.^30^ These findings have assisted with the selection of new centers and institutions with which to partner as GIPAP has expanded worldwide. These analyses, made possible through analysis of GIPAP data, address a critical deficit in the study of CML by providing data on treatment patterns and outcomes in LMICs that were largely unknown.

## DISCUSSION

In the past 14 years, GIPAP has provided approximately 2.3 million monthly doses of imatinib to more than 49,000 patients in LMICs, representing a large humanitarian effort of unprecedented scope and impact. One of the most significant findings throughout the evolution of GIPAP is that providing access to targeted cancer therapies can be a catalyst for the subsequent development of resources and improvement of health care systems, as opposed to the more common strategy of some sponsors in supporting capacity building efforts but excluding access to drugs. In addition, cooperation with key local stakeholders and alignment with local authorities at early stages should be sought. The model itself—partnering with an NGO such as The Max Foundation for tracking the treatment and drug consumption of each individual patient and offering wraparound patient support services—greatly contributes to overcoming shortfalls from bulk international humanitarian donations, minimizing drug diversion, and improving treatment compliance. Companies developing lifesaving medicines to treat noncommunicable diseases such as cancer should strongly consider implementing global access strategies with a patient-centered approach such as the one used for GIPAP. There will always be a role in the area of global oncology for international donations of drugs, and this program provides an example of how access to innovative cancer treatment in LMICs is both possible and beneficial to the global community as well as individual patients.

There are many known barriers to access to innovative cancer therapies in LMICs, affordability being commonly cited as one. In the GIPAP example, the program removes the variable of affordability; no quotas on the number of patients have ever been imposed by Novartis, and all applications submitted by approved physicians in GIPAP open countries have been and will continue to be evaluated on their own merit. Still, lack of access to diagnostics, favorable socioeconomic conditions, and viable health care infrastructure may prevent patients from accessing otherwise available treatments. As such, the program has shed light on the need for a broader approach to finding solutions to the complex causes of lack of access to treatment. Collectively, our findings from GIPAP are important, because the GIPAP population comes from countries where it is estimated that 70% of all new cancer diagnoses will be made^31^; the findings highlight that robust partnerships between NGOs and pharmaceutical companies for programs similar to GIPAP could serve as a tool to improve patient access, research, and outcomes. GIPAP is an example of how to effectively provide patient assistance in countries where little or no health insurance or prescription drug coverage exists. This model demonstrates that such initiatives can work in concert with existing local health care structures to treat life-threatening conditions worldwide.
